# Differential prolactin levels among male and female patients with thyroid related complains in the Hail regions of Saudi Arabia

**DOI:** 10.6026/97320630015633

**Published:** 2019-10-09

**Authors:** Gamal Mohamed Elawad Ahmed, Jerold Casem Alcantara, Samir Abdulkarim Alharbi, Waled Mansi Alshammari, Fawaz Dabae Alshammari, Ibtihag Siddig Elnaem, Abdelmuhsin Omer Ahmed Hassan, Khalid Sowilih Alrashedi, Ahmed Abdulaziz Al Tayyar, Turky Ali K Alreshidi

**Affiliations:** 1 Department of Clinical Laboratory Science, College of Applied Medical Sciences, University of Hail, Saudi Arabia; 2 Department of Clinical Laboratory Science, College of Applied Medical Sciences, Shaqra University, Saudi Arabia; 3 Hail King Khaled Hospital, Hail, Saudi Arabia; 4 College of Dentistry, University of Hail, Saudi Arabia; 5 Hail Regional Laboratory, Hail, Saudi Arabia

**Keywords:** FSH, hormones, luteinizing hormone, prolactin, T3, T4, thyroid disorders, TSH

## Abstract

Thyroid diseases are caused by autoimmunity due to hormone imbalance both in male and female patients. Therefore, it is of acute importance to measure, analyze and compare thyroid hormone levels among populations with thyroid-related complications. Hence, we examined 202 male and female thyroid patients in the Hail regions of Saudi Arabia and estimated their hormone levels. Blood samples were collected from patients and processed for the hormonal profiling such as prolactin, luteinizing hormone (LH), FSH, free T3 (FT3), free T4 (FT4) and TSH3. Further, measurement of thyroid gland size in the Hail population was also completed. Results of our study showed a significant difference in the level of prolactin between male and female patients. Other hormones are namely luteinizing hormone (LH), FSH, free T3 (FT3), free T4 (FT4), TSH3 did not show any significant difference between male and female patients with thyroid disorder. Thus, the levels of the majority of hormones, namely luteinizing hormone (LH), FSH, free T3 (FT3), free T4 (FT4), and TSH3, except prolactin, did not differ significantly between male and female thyroid patients. Validation of the observation using large scale population size is warranted in future investigations.

## Background

Thyroid hormones assume a vital job in the digestion of the human body. Changes in the thyroid organ action show in almost all body frameworks. Proper learning of general society about thyroid issues and their signs are essential for early recognition [1,2]. This contextual analysis is directed to explore the thyroid hormone levels and related issues, including assessment of dietary conditions in the rustic zone of hail area. Thyroid gland disorders are mainly caused by the autoimmunologic assault, either leading to thyroid hormone excess or deficiency. [3,4]. The thyroid gland produces stimulating thyroid hormone (TSH), total triiodothyronine (T3), free triiodothyronine (FT3), total thyroxine (T4), and free thyroxine (FT4) [5,6]. It has been reported that thyroxine (T4) is converted to T3 by removal of one iodine atom (I) from T4.[7]. Studies revealed that during development, both T3 and T4 play an essential role in cellular differentiation and maintain metabolic homeostasis. [8]. Among the above-secreted hormones from the thyroid glands, TSH is the most crucial marker for diagnosis of thyroid dysfunction [9]. TSH released in the circulation in response to thyrotropin-releasing hormone (TRH), directly secreted from the hypothalamus. Upon the release of TSH in the distribution, the synthesis and secretion of both thyroid hormone (T3 and T4) begin to start. To development and metabolism of virtually all tissues, the roles of thyroid hormones are much essential [10].The molecular signaling pathway of thyroid hormones is primarily mediated by the binding of the T3 (bioactive form) to the nuclear T3 receptor (Trs).Excessive release of TSH in the circulation results in the development of hyperthyroidism. It has been reported that hyperthyroidism itself develops a condition known as thyrotoxicosis. [11-13], which ultimately raised Graves'disease. Thyroid storm is another life-threatening condition where the level of thyroid hormones in the circulation was increased. Several reports revealed that women are most susceptible to thyroid storm than man [14], and the average ages of women for the occurrence of thyroid storms in between 20 to 49 years [14,15]. Hyperthyroidism itself induces several other disease conditions [16]. Studies reported that patients with hyperthyroidism have a higher incidence of β-cell dysfunction leading to insulin resistance and exacerbating hyperglycemia. [16,17].18% of thyroid deaths are due to thrombogenic complications because of the higher level of blood coagulation factor VIII, IX, and VWF [18]. The most widely recognized causes of hyperthyroidism are Graves' sickness and dangerous nodular goiter. What's more, it might result from thyroiditis or ingestion of abundance thyroid hormones, what's more, it might, once in a while, be iodine-incited. Treatment choices change as indicated by the case, and they may incorporate antithyroid medications, β blockers, radioactive iodine treatment, and medical procedures. Thyroid tempest is a unique hazardous condition that needs a careful appraisal and careful treatment [19-24]. A few contemplate from various nations' revealed high pervasiveness of thyroid sicknesses with higher rates of hypothyroidism than hyperthyroidism. Both plain and Appraisal of Public Knowledge. In Saudi Arabia, thyroid dysfunctions are the most widely recognized endocrine issue. Also, thyroid malignancy was announced as the second most regular malignant growth among Saudi females with strikingly high occurrence in the Hail locale [25-28]. We report herein the serum levels of prolactin, luteinizing hormone (LH), FSH, free T3 (FT3), free T4 (FT4), TSH3 in male and female patients suffered from a thyroid disorder. We found that there is a significant difference in the level of prolactin only between male and female patients. Consequently, the present investigation was led to evaluate open learning concerning the distinctions among hyperthyroidism and hypothyroidism in Hail, Saudi Arabia.

## Methodology

### Ethical consideration:

Members were educated about the examination goals and methodology. Subjects, who consented to fill the survey, suggested that they allowed taking part in the examination. The investigation didn't demonstrate any physical, mental, social, lawful, financial, or some other dangers to the investigation's members. The research monitored members' protection. Examiners were in charge of keeping the security of the information. All members' information was not utilized for some other reason outside this examination.

### The study was done after approval of the ethical board of the University of Hail:

Study design: This is a descriptive study conducted in a regional laboratory in the hail region. This is a cross-sectional examination.

### Study Population: 

All patients referred to the laboratory for thyroid-related complains during the study period were includes N-302.

### Patients

The current comparative study included three hundred two thyroid patients (n = 302). The mean age of the patients was 36.88 ± 0.78.

### Sample collection and preparation

Blood samples (volume = 3ml) were collected from all the patients and were stored in plain glass/ plastic vials for at least one hour at room temperature. After that, all the samples were centrifuged at 3000 to 3500 rpm for 5 minutes. After centrifugation supernatant (serum) was transferred into fresh 5 ml polypropylene vials, all samples were kept at ambient temperature (20-25°C) to protect the samples from the possible evaporation effects. Samples were processed for further analysis within 2 hours.

### Determination of prolactin in serum samples of male and female patients

Prolactin was measured by electro chemi luminescence immunoassay (ECLIA) intended for use on Elecsys and cobras e immunoassay analyzers. This method is for the quantitative determination of prolactin in human serum and plasma. The plan is based on the sandwich principle. At first incubation, ten µL of sample and a biotinylated monoclonal prolactin specific antibody form the first complex. A second incubation: After the addition of a monoclonal prolactin-specific antibody labeled with a ruthenium complex and streptavidin-coated microparticles, a sandwich complex is built and becomes bound to the solid phase via interaction of biotin and streptavidin. The reaction mixture is aspirated into the measuring cell where the microparticles are magnetically captured onto the surface of the electrode. Unbound substances are then removed with ProCell/ProCell M. Application of a voltage to the wire then induces chemiluminescent emission which is measured by a photomultiplier. Results are determined via a calibration curve, which is an instrument generated explicitly by 2-point calibration and a master curve provided via the reagent barcode or e-barcode.

### Determination of FSH and LH in the serum samples of male and female patients

FSH was measured by immuno radiometric assay. Briefly, the method includes the coating of monoclonal anti-FSH antibody inside of a polystyrene assay tube, and endogenous FSH is bound to the immobilized monoclonal antibody 1. I125 anti-FSH monoclonal antibody two was added, and a sandwich was formed with the endogenous FSH at the center. Excess reagents were washed away, and the radioactivity bound was measured. The amount of radiation is proportional to the amount of hormone present in the sample [21].male and female. Data represent in mean ± S.D. * p < 0.05, ** p < 0.001. (F) Serum TSH levels of male and female. Data represent in mean ± S.D. * p < 0.05, ** p < 0.001.

LH was measured by electro chemi luminescence immunoassay "ECLIA" intended for use on Elecsys and Cobas e immunoassay analyzers. This method is for the quantitative determination of prolactin in human serum and plasma. The process is based on the sandwich principle. At 1st incubation, 20 µL of the sample, a biotinylated monoclonal LH-specific antibody, and a monoclonal LH-specific antibody labeled with a ruthenium complex form a sandwich complex. At 2nd incubation, after the addition of streptavidin-coated microparticles, the compound becomes bound to the solid phase via interaction of biotin and streptavidin. After that, the reaction mixture is aspirated into the measuring cell where the microparticles are magnetically captured onto the surface of the electrode. Unbound substances are then removed with ProCell/ProCell M. Application of a voltage to the wire then induces chemi luminescent emission which is measured by a photomultiplier. Results are determined via a calibration curve, which is an instrument generated explicitly by 2-point calibration and a master curve provided via the reagent barcode.

### Determination of free T3 (FT3) and free T4 (FT4) in serum samples of male and female patients

Serum samples were thawed at room temperature. After that, 400 µL of serum sample was placed in a 30 kDa ultrafiltration device (Centrifree YM-30, Millipore) and centrifuged in at 2700 rpm at 37°C for 30 min. Next, 150 µL of ultrafiltrate mixed with 450 µL methanol containing internal standards, deuterium-labeled T4, and carbon-labeled T3 for deproteinization. Vortex the mixture for the 30s and centrifuged for 10 min at 13,000 rpm. Next, 500 µL of supernatant was diluted with 600 µL of distilled de-ionized water, and 400 µL of an aliquot from them was injected onto a Phenomenex Kinetex 2.6 µm C18 column (73 x 2.2 mm). After washing, the analyze compounds were eluted from the column with a water/methanol gradient into the MS/MS system [21].

### Determination of TSH in serum samples of male and female patients

The immunoradiometric assay using I135 measured the FSH level in the serum. Briefly, excess monoclonal-labeled TSH antibody was mixed with serum samples. After equilibrium, a solid-phase TSH antibody was added, and the reaction was again allowed for completion. At this moment, TSH bound to labeled TSH antibody is also linked to a solid-phase antibody, which provides for separation of TSH with its conjugated antibodies and labels from the reaction mixture [13].

### Statistical analyses

Data analysis was carried out using SPSS version 22 and GraphPad Prism. Categorical variables were summarized as frequencies and percentages, and an association between variables was tested using the Chi-Square test. t-test was employed to compare the means of all parameters between the two groups, i.e., male and female. Factors were considered significantly different at a level p ≤0.05.

## Results

### Comparison in the level of serum prolactin in male and female patients

**Figure 1:**
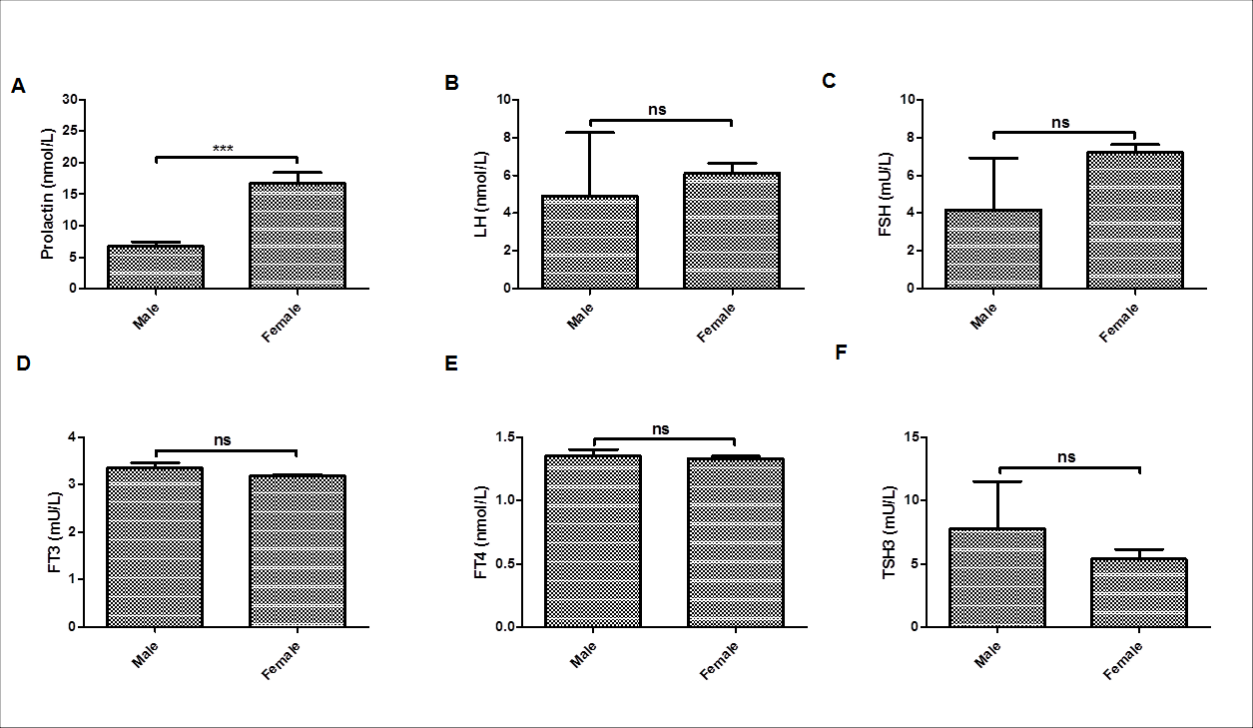
(A) Serum Prolactin levels of male and female. Data represent in mean ± S.D. * p < 0.05, ** p < 0.001. (B) Serum LH levels of male and female. Data represent in mean ± S.D. * p < 0.05, ** p < 0.001. (C) Serum FSH levels of male and female. Data represent in mean ± S.D. * p < 0.05, ** p < 0.001. (D) Serum FT3 levels of male and female. Data represent in mean ± S.D. * p < 0.05, ** p < 0.001. (E) Serum FT4 levels of male and female. Data represent in mean ± S.D. * p < 0.05, ** p < 0.001. (F) Serum TSH levels of male and female. Data represent in mean ± S.D. * p < 0.05, ** p < 0.001.

Results of serum prolactin in male and female patients are represented in Table 1 and Figure 1. Results revealed that there is a significant difference in the level of serum prolactin in male and female patients (t = -5.37; df = 62.81; p ≤ 0.001 (Table 1 and Figure 1). The level of prolactin was lower in male patients as compared to female patients. Male have mean prolactin level of 6.77 nmol/L, while females have mean prolactin level of 16.73 nmol/L (Table 1 and Figure 1a).

### Comparison in the level of serum LH in male and female patients

Results of serum LH in male and female patients are represented in Table 1 and Figure 1. Results revealed that there is no significant difference in level of serum LH in male and female patients (t = -0.35; df = 1.04; p = 0.80) (Table 1 and Figure 1). However, the level of LH was lower in male patients as compared to female patients. Male have mean LH level of 4.90 nmol/L, while as females have mean LH level of 6.11 nmol/L (Table 1 and Figure 1b).

### Comparison in the level of serum FSH in male and female patients

Results of serum FSH in male and female patients are represented in Table 1 and Figure 1). Results revealed that there is no significant difference in level of serum FSH in male and female patients (t = -1.09; df = 1.05; p = 0.46) (Table 1 and Figure 1c). However, the level of FSH was lower in male patients as compared to female patients.Male have mean FSH level of 4.18 mU/L, while as females have mean FSH level of 7.19 mU/L (Table 1 and Figure 1c).

### Comparison in the level of serum free T3 (FT3) and free T4 (FT4) in male and female patients

Results of serum FT3 and FT4 in male and female patients are represented in Table 1 and Figure 1d;Figure 1e). Results revealed that there is no significant difference in level of serum FT3 (t = 1.48; df = 47.66; p = 0.14) and FT4 (t = 0.40; df = 50.77; p = 0.69) in male and female patients (Table 1 and Figure 1 and Figure 1 ). However, the levels of FT3 and FT4 were slightly higher in male patients as compared to female patients. Male have mean FT3 level of 3.35 mU/L, while as females have mean FT3 level of 3.18 mU/L (Table 1 and Figure 1d and Figure 1e). Similarly, Male have mean FT4 level of 1.35 nmol/L, while as females have mean FT4 level of 1.33 nmol/L (Table 1 and Figure 1d and Figure 1e).

### Comparison in level of serum TSH3 in male and female patients

Results of serum TSH3 in male and female patients are represented in Table 1 and Figure 1f). Results revealed that there is no significant difference in level of serum TSH3 in male and female patients (t = 0.63; df = 42.30; p = 0.53) (Table 1 and Figure 1f). However, the level of FSH was higher in male patients as compared to female patients. Male have mean FSH level of 77.80 mU/L, while as females have mean FSH level of 5.38 mU/L (Table 1 and Figure 1f).

### Association of age and clinical parameters with sex

Chi Square test revealed a significant association between age and sex (χ (4) = 1 15.074, p ≤ 0.01) only, while as other parameters such as TSH3 ( χ(2) = 0.941, p = 0.625); FSH (χ (3) = 1.406, p = 0.704); FT3 (χ (1) = 0.642, p = 0.423); FT4 (χ (4) = 2.762, p = 0.251); PRL (χ (2) = 1.177, p = 0.555) did not showed any significant association with sex.

## Discussion

Thyroid hormones assume a crucial job in the digestion of the human body. Changes in the thyroid organ action show in almost all body frameworks. Proper learning of general society about thyroid Disarranges and their signs are essential for right on time recognition. The point of the study: to survey open information concerning contrasts among hyperthyroidism and other parameters in the Hail region of Saudi Arabia. To the fussiest of the creators' learning, this is the primary investigation to explore this point. The most widely recognized reason for essential thyroid illness is dietary iodine insufficiency. Iodine is a crucial segment in the structure of thyroid hormones. Other basic rights for essential thyroid illness incorporate immune system devastation (Hashimoto's thyroiditis), radiation-prompted thyroiditis, postsurgical hypothyroidism, and thyroid medications, and infiltrative illness [29]. Innate components influence the weakness to create thyroid issue. Hereditary qualities play an outstanding job in both assurances of thyroid hormone and Thyrotropin (TSH) focuses, and vulnerability to immune system thyroid infection [30]. Every single thyroid issue is more typical in ladies than men. Hyperthyroidism was found in a more significant number of ladies than men (5:1 proportion).

In this study, is a comparative study involving two groups (male and female) thyroid patients. We report herein the serum levels of prolactin, luteinizing hormone (LH), FSH, free T3 (FT3), free T4 (FT4), TSH3 in male and female patients suffered from a thyroid disorder. Thyroid hormones in the system play an essential role in the balance between over producing ROS and antioxidant defence. It has been reported that thyroid hormones modulating various aspects of nervous system development [15]. In neurological diseases, thyroid hormones act as a critical and important regulator for regeneration following neuro injury [16]. The action of thyroid hormone was mediated by repairing intracellular H+ accumulation and increased the levels of several neuro protective proteins [17]. The results of the present study showed that there is a significant difference only in the level of prolactin between male and female patients. Other hormones namely luteinizing hormone (LH), FSH, free T3 (FT3), free T4 (FT4), TSH3 did not show any significant difference between male and female patients suffered from thyroid disorder. Similar kinds of findings were reported from different studies conducted across the globe [13].

However, other studies have documented that the levels of these hormones may be altered in other diseases. It has been reported that patients suffering from multiple sclerosis have high level of TSH and serum T3 and T4 but the serum concentration of TSH is more sensitive than T3 and T4 for the disease pathogenesis. Studies revealed that there is a synergistic relation between thyroid hormones and glucagon on lipid metabolism and body weight. The critical role of thyroid hormones on systemic metabolism largely depends on glucagon action. Glucagon acts as a targeting ligand for thyroid hormone action in liver and adipose tissue. It has been reported that both glucagon and thyroid hormones have individual important effects on liver triglyceride metabolism by glucagon mediated targeting T3 [18], which effectively removes fat deposition in liver. This synergistic signaling pathway also crucial for liver cholesterol uptake and cholesterol metabolism. Study with obese mice, it was found that glucagon mediated targeting T3 is very much effective for weight loss, due to increased energy expenditure via lipolytic mechanism [19]. However, in another study, it was reported that in euthyroid obese individuals, there are no association between energy expenditure and serum TSH, T3 and T4 levels [20]. The plausible explanation for this in the impairment of thyroid hormone action in the adipose tissue of those obese individuals due lack of thyroid hormone receptors [21]. Results also revealed a significant association between age and sex only, while as other parameters such as TSH3, FSH, FT3, FT4, PRL did not showed any significant association with sex. For comparison of these results we could not found any specific study. The results of the current study, we conclude that the levels of the majority of hormones namely luteinizing hormone (LH), FSH, free T3 (FT3), free T4 (FT4) and TSH3, except prolactin, did not differ significantly between male and female thyroid patients. Therefore, the findings of our study cannot be generalized. For the validation of our results, an investigation with large sample size is warranted.

## Conclusion

Data on the thyroid hormone levels among male and female populations with thyroid-related complications in the Hail regions of Saudi Arabia is highly relevant in diagnosis and treatment. We report the differential prolactin levels among male and female patients with thyroid-related complains in the Hail regions of Saudi Arabia. However, this is not true for hormones such as luteinizing hormone (LH), FSH, free T3 (FT3), free T4 (FT4) and TSH3. Validation of the observation using large scale population size is necessary in future investigations for further confirmation.

## Figures and Tables

**Table 1:** Comparison of prolactin, LH, FSH, FT3, FT4, and TSH3 between male and female thyroid patients

Variable	Male subjects	Female subjects	t value; df; p value
Prolactin (nmol/L)	6.77± 0.65*	16.73 ± 1.73	-5.37; 62.81; <0.001
LH (nmol/L)	4.90 ± 3.36	6.11 ± 0.52	-0.35; 1.04; 0.80
FSH (mU/L)	4.18 ± 2.73	7.19± 0.43	-1.09; 1.05; 0.46
FT3 (mU/L)	3.35 ± 0.11	3.18 ± 0.03	1.48; 47.66; 0.14
FT4 (nmol/L)	1.35± 0.05	1.33 ± 0.01	0.40; 50.77; 0.69
TSH3 (mU/L)	7.80 ±3.71	5.38 ± 0.75	0.63; 42.30; 0.53
*Values are mean ± 1 SEM

## References

[1] Aakre I (2017). PloS one.

[2] Kelley ST (1974). Vet Med Small Anim Clin.

[3] Antonelli A (2015). Autoimmun Rev.

[4] Mooradian AD (2019). Drugs Aging.

[5] Pimentel L (2005). Emerg Med.

[6] Warnet A (1992). Metabolism.

[7] Braverman LE (1970). The Journal of clinical investigation.

[8] McAninch EA (2014). Ann N Y Acad Sci.

[9] Spencer CA (1996). Clin Chem.

[10] Fraichard A (1997). EMBO J.

[11] Davies TF (2005). J Clin Invest.

[12] Mio C (2019). Clin Genet.

[13] Burch HB (2019). N Engl J Med.

[14] Hampton J (2013). AACN Adv Crit Care.

[15] Szpunar MJ (2018). Arch Womens Ment Health.

[16] Mayer-Roenne B (2007). Journal of feline medicine and surgery.

[17] Scott KG (1960). Cancer.

[18] Clagett GP (1974). JAMA.

[19] Bartalena L (2013). Nature Reviews Endocrinology.

[20] Salem JE (2019). JAMA cardiology.

[21] Surks MI (1988). J Clin Endocrinol Metab.

[22] Hopton MR (1986). Clin Chem.

[23] Denenberg VH (1958). J Comp Physiol Psychol.

[24] Denenberg VH (1958). J Comp Physiol Psychol.

[25] Bazarbashi S (2017). Asian Pacific journal of cancer prevention: APJCP.

[26] Kalra S (2016). Indian J Endocrinol Metab.

[27] Lotufo PA (2016). Sao Paulo Medical Journal.

[28] Almutairi FM (2017). Saudi Journal of Kidney Diseases and Transplantation.

[29] Ai J (2003). Journal of the American Academy of Dermatology.

[30] Panicker V (2011). Clin Biochem Rev.

